# Highlights of Keystone symposium ‘Fibrosis: from bench to bedside’

**DOI:** 10.1186/1755-1536-7-11

**Published:** 2014-08-15

**Authors:** Judith Jeanette de Haan, Fatih Arslan

**Affiliations:** 1 Universiteit Medisch Centrum, Heidelberglaan 100 3584 CX, Utrecht, The Netherlands

**Keywords:** Fibrosis, Keystone, Myofibroblast, TGFβ

## Abstract

This report is based on the ‘Fibrosis: from bench to bedside’ symposium from the Keystone Symposia meeting series, Keystone, Colorado, 23 to 28 March 2014. It was a fascinating symposium with high quality talks, workshops, and well attended poster sessions.

## Introduction

Fibrosis occurs in many organs upon injury or due to genetic causes. The pathological bases of various fibrotic diseases share considerable overlap with each other. An inflammatory reaction is initiated to remove debris after any kind of injury that results in cell death. In the meantime, fibroblasts or other myofibroblast pre-cursors are attracted to the site of injury where they start to proliferate and secrete extracellular matrix (ECM). After making a collagen-based scar, the myofibroblasts will either become apoptotic or go into senescence. During this symposium, various fibrotic processes were discussed and both old and new players in fibrosis were debated.

### Overview of selected talks

Harry Dietz (John Hopkins University School of Medicine, USA) kicked off the symposium with an interesting talk on scleroderma and systemic sclerosis (SSc) and stiff skin syndrome (SSS). SSc is an autoimmune disease in which the skin and other organs undergo fibrotic changes. Harry Dietz used a fibrillin-1 knock-out (KO) mouse model to study SSc and SSS. Fibrillin-1 KO mice display the same phenotype as patients with SSS, and the effects observed are regulated by integrin signaling, because this phenotype is prevented by integrin-modulating therapies. Fibrillin-1 is also known to regulate TGFβ availability. (Chaudhry *et al.*, Journal of Cell biology, 2007) The group of Harry Dietz observed a 40-fold increase in TGFβ activity and that a TGFβ neutralizing antibody prevented fibrosis. A potential regulator of the above mentioned fibrotic changes might be microRNA29a (miR29a). Nelson Chau (Regulus Therapeutics, San Diego, CA, USA) spoke more about the emerging role of miRs in fibrosis. miR29 is known to suppress ECM production. Furthermore, miR29 is downregulated in patients with cirrhosis and can therefore be used as a biomarker.

Judith Campisi (Buck Institute for Research on Aging, Novato, CA, USA) continued with a talk on senescent cells and their role in wound healing. Senescent cells secrete senescence-associated secreted phenotype (SASP), which includes numerous proteins, cytokines and matrix metalloproteases (MMPs). In turn, SASP inhibits stem cell proliferation. Transient presence of senescent cells is required for proper wound healing. Eliminating senescent cells results in highly fibrotic wounds. However, in diseases associated with aging, it appears that senescent cells are continuously present, which can lead to a deleterious phenotype.

Currently, a lot of research is being conducted on pericytes and their ability to transdifferentiate into myofibroblasts. Jeremy Duffield (University of Washington, USA) discovered a Foxd1 and PDGFRβ positive cell lineage that can become myofibroblasts upon disease. Lucie Peduto (Institut Pasteur, France) identified Gp38 + ADAM12+ stromal cells that are present after injury. ADAM12 is induced by TGFβ, and ADAM12 positive pericytes infiltrate into the tissue upon injury and disappear again when the injury is resolved. In the case of chronic injury, the cells settle in the tissue but lose their ADAM12 expression. In the absence of ADAM12, there is less production of collagen after injury. Christian Göritz (Karolinska Institutet, Sweden) talked about a subset of pericytes that is involved in the repair of lesions of the central nervous system called type A pericytes. When 80% of type A pericytes are eliminated it will reduce scar formation. Total depletion of type A pericytes results in defective healing of the lesion.

An interesting talk by David Lagares (Massachusetts General Hospital and Harvard Medical School, USA) showed that tissue stiffness controls cell fate; fibroblasts stay at 0.5 kPa, while myofibroblasts are present in tissues with 25 kPA. Stiffness is not evenly distributed within tissue, and they show that fibroblasts move towards a stiffer environment. This process is called durotaxis. In a stiffer environment, the fibroblasts will transdifferentiate and start to express αSMA and secrete ECM proteins. This process is called fibronucleation. It creates a vicious cycle wherein an already stiff environment attracts and activates fibroblasts.

Massimiliano Mellone (University of Southampton, UK) described the role of DNA damage and DNA damage response (DDR) pathways in myofibroblast transdifferentiation. DNA damage leads to senescent fibroblasts and that these senescent fibroblasts start to express αSMA. TGFβ can activate DDR pathways and also induce senescence. Furthermore, when you inhibit DDR pathway, less myofibroblast transdifferentiation takes place. In line with Massimiliano Mellone’s talk, Neetu Razdan (Rutgers University, NJ, USA) discussed the role of telomere dysfunction in αSMA expression. Prolonged culturing of fibroblasts results in telomere shortening, a naturally occurring phenomenon associated with aging. Aging fibroblasts increase their αSMA expression. Inhibiting telomere shortening by adding hTERT results in reduced αSMA expression.

David Brenner (University of California, San Diego Health Sciences, CA, USA) addressed the question of where myofibroblasts go after repair in the injured liver. He showed that 50% go into apoptosis while the other 50% turn into quiescent hepatic stellate cells (HSC), one of the major pools for myofibroblasts in the liver. These quiescent HSC are not the same as inactivated HSC, as is shown by gene profiling. Additionally, quiescent HSC seem to have a “memory for injury” as they are more rapidly reactivated than inactivated HSC.

Autophagy, a catabolic state the cell uses to maintain viability under stress conditions, is involved in many processes, including fibrosis. Monique Bernard (University of Montreal Hospital Centre, Canada) linked autophagy to myofibroblast transdifferentiation. In addition, Scott Friedman (Mount Sinai School of Medicine, NY, USA) explained that autophagy takes place in stellate cells upon fibrotic stimuli. Deficiency of Atg7FP, an autophagy gene, leads to proliferation through Yes-Associated Protein (YAP), a protein known to play a role in Hippo-signaling-mediated activation of fibroblasts along with the protein TAZ (Transcriptional coactivator with PFZ-binding motif). James Pritchett (University of Manchester, UK) showed that HSCs get activated when cultured on plastic and start to express αSMA which leads to an increased expression of YAP/TAZ. Knocking out YAP leads to a reduction of αSMA expression and also to a reduction of Sox9, which is known to regulate ECM expression during development.

### Translation to the clinic

It is of great clinical importance to have a method for early detection of fibrosis. As mentioned above, miR29 could be a new biomarker for liver fibrosis. In addition, Timothy Radstake (University Medical Center Utrecht, The Netherlands) presented data on the increased levels of CXCL4 in SSC patients and how this can be a novel biomarker, linking inflammation, endothelial dysfunction and fibrosis together. In addition to using fibrosis for the detection of disease, it can also be targeted with new medication. For example, Jeffrey Crosby (ISIS Pharmaceuticals, Carlsbad, CA, USA) suggested using antisense oligonucleotides for osteopontin (OPN) for the treatment of idiopathic pulmonary fibrosis (IPF). Using these oligonucleotides decreases OPN levels in macrophages in the bronchoalveolar lavage from mice and also reduces the damage in their lungs.

Elizabeth Trehu (Promedior, Inc., MA, USA) and Bernt van den Blink (Erasmus MC, Netherlands) showed that PRM-151, a recombinant form of Pentaxin-2 which regulates monocyte differentiation and can decrease fibrosis, increases forced vital capacity in patients with IPF. Moreover, PRM-151 has already been shown to be effective in animal models of lung, kidney, skin, and heart fibrosis. Cédric Szyndralewiez (Genkyotex, Switserlands) presented data on GKT137831, an NAPDH oxidase (NOX) inhibitor. NOX1/4 plays an important role in liver fibrosis by inducing apoptosis, myofibroblast differentiation, and infiltration of macrophages. GKT137831 reduced fibrosis in different liver fibrosis models by reducing the infiltration of macrophages.

Dean Sheppard (University of California, CA, USA) talked about the possibility of using the integrin αv receptor on fibroblasts as a therapeutic target to prevent fibrosis. He showed that deletion of fibroblast integrin αv prevents fibrosis in a hepatic, renal, and pulmonary model. Furthermore, a small molecule inhibitor against αvβ1 inhibited the formation of fibrosis in bleomycin-induced pulmonary fibrosis and CCl_4_-induced hepatic fibrosis, suggesting that this is a promising new drug.

Benjamin Alman’s PhD student (Duke University, NC, USA) presented data from a high throughput screen which found nefopam to be a β-catenin inhibitor which could prevent fibrosis. Topical 1% nefopam cream decreased skin wound size in biopsy wounds in mice and pig models.

Robert Lafyatis (Boston University School of Medicine, MA, USA) found that fresolimumab, a thrombospondin neutralizing antibody, improved the clinical Rodnan score in SSc patients. On top of that, ADAM12 levels predicted the improvement in thrombospondin levels and might be a new biomarker for SSc.

**Figure 1:**
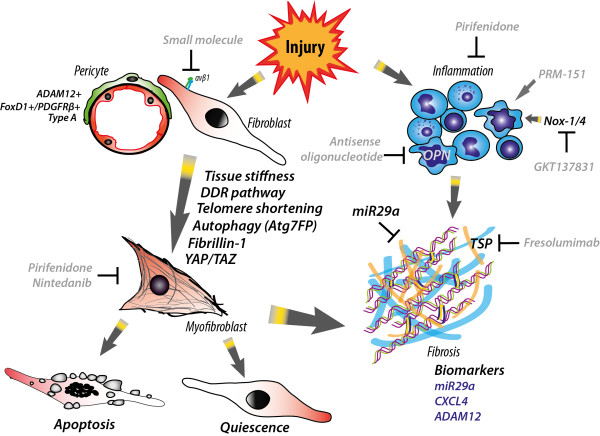
**Highlights of the pathophysiology of fibrosis.** Injury will lead to both activation of the immune system as well as activation of pericytes and fibroblasts. Pericytes and fibroblasts can differentiate into myofibroblassts and many factors, listed here next to the arrow, have an influence on this process. Myofibroblasts contribute to the secretion and remodeling of extracellular matrix, thereby inducing fibrosis. miR29a can inhibit fibrosis. Inflammation also has a role in injury and subsequent fibrosis. Attracted leukocytes clear up debris and secrete inflammatory molecules. A lot of factors can influence the inflammatory response, such as NOX-1/4 which is known to be involved in the influx of macrophages. When the damage is repaired and a fibrotic scar is formed, myofibroblasts can either go into apoptosis or become quiescent fibroblasts. Biomarkers for this fibrotic mechanism are listed in purple. Furthermore, there are current preclinical and clinical trials trying to inhibit fibrosis. The molecules and drugs from these trials are shown in grey.

Importantly, in 2014, two large, randomized, placebo controlled Phase III trials were completed. Paul Noble (Cedars-Sinai Medical Center, CA, USA) presented the outcome of these trials: The ASCEND trial (pirfenidone) and INPULSIS (nintedanib) study. Both pirfenidone and nintedanib reduced the decline of forced vital capacity over a 52-week period. These drugs show great potential for patients with IPF.This overview (summarized in Figure 1) discussed just a small selection of all the great talks that were presented at this symposium. The program of this symposium was perfect to intermingle and to discuss new and exciting data with each other. This was a very successful symposium and many great ideas and collaborations will certainly arise from this week.

## Competing interests

The authors declare that they have no competing interests.

## Authors’ contributions

JJH wrote the manuscript. FA helped to draft the manuscript. Both authors read and approved the final manuscript.

